# Supplementary material for: Phenotypic, Technological, Safety, and Genomic Profiles of Gamma-Aminobutyric Acid-Producing Lactococcus lactis and Streptococcus thermophilus Strains Isolated from Cow’s Milk
Source: Int J Mol Sci. 2024 Feb 16;25(4):2328. doi: 10.3390/ijms25042328 (PMC10889254; doi:10.3390/ijms25042328)
Supplement: Supplementary file 1 [file ijms-25-02328-s001.zip › ijms-2845902-supplementary.pdf]

Table S1. Carbohydrate fermentation profile of the *Lactococcus lactis* and *Streptococcus thermophilus* GABA producer strains of this study.

Species/ strain	Carbohydrate[a]																				
	ARA	RIB	XYL	GAL	GLU	FRU	MAN	MNN	NAG	AMY	ARB	ESC	SAL	CEL	MAL	LAC	SUC	TRE	STA	GEN	GNT
L. lactis																					
Lc 5.5	-	+	+	+	+	+	+	+	+	+	+	+	+	+	+	+	+	+	+	+	+
Lc 14.4	+	+	+	+	+	+	+	+	+	+	+	+	+	+	+	+	+	+	+	+	+
Lc 19.3	-	+	+	+	+	+	+	+	+	+	+	+	+	+	+	+	-	+	+	+	+
Lc 21.1	-	+	-	+	+	+	+	-	+	-	+	+	+	+	-	+	-	+	-	+	-
S. thermophilus																					
St 8.1	-	-	-	+	+	(+)	-	-	-	-	-	-	-	-	-	+	+	-	-	-	-
St 9.1	-	-	-	-	+	-	-	-	-	-	-	-	-	-	-	+	+	-	-	-	-
St 18.1	-	-	-	+	+	-	-	-	-	-	-	-	-	-	-	+	+	-	-	-	-
St 21.1	-	-	-	-	+	-	-	-	-	-	-	-	-	-	-	+	+	-	-	-	-

[a]Key of utilized carbohydrates: ARA L-arabinose; RIB, D-ribose; XYL, D-xylose; GAL, D-galactose; GLU, D-glucose; FRU, D-fructose; MAN, D-mannose; MNN, D-mannitol; NAG, N-acetylglucosamine; AMY, amygdalin; ARB, arbutin; ESC, esculin; SAL, salicin; CEL, D-cellobiose; MAL, D-maltose; LAC, lactose; SUC, D-sucrose; TRE, D-trehalose; STA, starch; GEN, gentiobiose, GNT, gluconate. In parenthesis, delayed reaction (weak utilization).

None of the strains utilized the carbohydrates glycerol, erythritol, D-arabinose, L-xylose, D-adonitol, methyl-β-D-xylopyranoside, L-sorbose, L-rhamnose, dulcitol, inositol, D-sorbitol, methyl-α-D-mannopyranoside, D-melibiose, inulin, D-melezitose, D-raffinose, glycogen, xylitol, D-turanose, D-lyxose, D-tagatose, D-fucose, D-arabitol, L-arabitol, 2-ketogluconate, and 5-ketogluconate.

Table S2. Enzymatic activities of GABA-producing *Lactococcus lactis* and *Streptococcus thermophilus* strains by the API-ZYM system.

Strain	Enzymatic activity[a]																	
	Al-Phos	Est	Est-Lip	Lip	Leu-Ary	Val-Ary	Cys-Ary	Try	α-chy	Phos	N-nph	α-gal	β-gal	β-gln	α-glu	β-glu	N-a-β-glu	α-man
L. lactis																		
**Lc 5.5**	5	5	5	5	>40	-	20	5	5	>40	>40	-	-	5	5	>40	-	5
**Lc 14.4**	5	-	10	5	>40	-	5	5	5	>40	>40	-	5	-	-	>40	-	-
**Lc 19.3**	5	5	5	5	>40	-	20	5	5	>40	>40	-	-	5	-	-	-	-
**Lc 21.1**	5	5	5	5	>40	-	20	5	5	30	30	-	-	5	-	-	-	-
S. thermophilus																		
**St 8.1**	-	-	-	5	>40	20	-	-	-	-	>40	-	>40	-	-	-	-	-
**St 9.1**	5	20	5	10	>40	20	5	5	5	5	>40	5	>40	5	-	-	5	-
**St 18.1**	-	20	-	10	>40	20	-	-	-	-	>40	-	>40	-	-	-	-	-
**St 21.1**	-	20	-	10	>40	20	-	-	-	-	>40	-	>40	-	-	-	-	-

[a]Activity: nmol substrate hydrolysed. Key of enzymes: Al-Phos, Alkaline phosphatase; Est, esterase (C8); Est-Lip, esterase-lipase (C14); Lip, Lipase (C 14); Leu-Ary, leucine arylamidase; Val-Ary, valine arylamidase; Cys-Ary, Cystine arylamidase; Try, Trypsin; α-chy, α-chymotrypsin; Phos; acid phosphatase; N-nph, naphtol-AS-BI-phosphohydrolase; α-gal, α-galactosidase; β-gal, β-galactosidase; β-gln, β-glucuronidase; α-glu, α-glucosidase; β-glu, β-glucosidase; N-a-β-glu, N-acetyl-β-blucosaminidase and α-man, α-mannosidase. None of the strains presented α-fucosidase activity.

-, no activity.

Table S3. Minimum inhibitory concentration (MIC) of 16 antibiotics to GABA-producing *Lactococcus lactis* and *Streptococcus thermophillus* from milk.

Species/strain	Minimum inhibitory concentration (µg mL^{-1})															
	**Gm**	**Km**	**Sm**	**Nm**	**Tc**	**Em**	**Cl**	**Cm**	**Am**	**Pc**	**Va**	**Q-da**	**Lz**	**Tm**	**Ci**	**Rif**
L. lactis																
**Lc 5.5**	1	8	32	4	0.5	0.12	0.06	8	0.5	0.5	0.25	2	4	>64	4	16
**Lc 14.4**	2	8	16	8	0.5	0.12	0.5	8	0.5	0.5	0.5	4	4	>64	4	16
**Lc 19.3**	1	8	16	16	0.12	0.06	0.12	4	0.5	0.5	0.25	4	2	>64	2	16
**Lc 21.1**	<0.5	8	16	1	1	0.12	0.06	4	0.25	0.25	0.5	2	2	>64	2	>64
**EFSA´s cut-offs[a]**	**32**	**64**	**32**	**-**	**4**	**1**	**1**	**8**	**2**	**-**	**4**	**-**	**-**	**-**	**-**	**-**
S. thermophilus																
**St 8.1**	2	16	8	2	0.5	0.12	0.03	2	0.12	0.12	1	0.25	2	64	2	0.12
**St 9.1**	8	**128**	32	32	1	0.06	0.03	4	0.25	0.5	0.5	0.25	2	64	2	0.25
**St 18.1**	1	16	8	4	1	0.25	0.03	4	0.12	0.12	1	0.25	2	64	2	0.12
**St 21.1**	2	16	8	4	0.5	0.12	0.03	4	0.12	0.5	0.25	0.12	2	>64	4	0.12
**EFSA´s cut-offs[a]**	**32**	**-**	**64**	**-**	**4**	**2**	**2**	**4**	**2**	**-**	**4**	**-**	**-**	**-**	**-**	**-**

Key of antibiotics: Gm, gentamicin; Km, kanamycin; Sm, streptomycin; Nm, neomycin; Tc, tetracycline; Em, erythromycin; Cl, clindamycin; Cm, chloramphenicol; Am, ampicillin; Pc, penicillin G; Va, vancomycin; Q-da, quinupristin-dalfopristin; Lz, linezolid; Tm, trimethoprim; Ci, ciprofloxacin; Rif, rifampicin.

[a]The cut-offs applied were those of EFSA (EFSA FEEDAP Panel, 2018).

-, cut-off not established.

Table S4. Genomic distance analysis of the *Lactococcus lactis* (A) and *Streptococcus thermophilus* (B) strains of this study with type strains of related species. Results from digital DNA-DNA hybridization (dDDH) are shown below the self-comparison diagonal, and results from average nucleotide identity (OrthoANI) are shown above the diagonal.

**A** ANI / dDDH	**Lc 5.5**	**Lc 14.4**	**Lc 19.3**	**Lc 21.1**	*L. lactis* subsp. *lactis* ATCC 19435^{T}	*L. lactis* subsp. *lactis* biovar. *diacetylactis* GL2^{T}	*L. cremoris* NBRC 100676^{T}	*L. cremoris* subsp. *tructae* DSM 21502^{T}	*L. allomyrinae* 1JSPR-7^{T}	*L. hircilactis* DSM 28960^{T}
**Lc 5.5**		**97.5**	**99.2**	**98.5**	**98.6**	**98.5**	86.9	86.5	76.1	73.2
**Lc 14.4**	**74.5**		**97.6**	**97.4**	**97.3**	**97.4**	87.0	86.5	75.8	73.0
**Lc 19.3**	**92.6**	**78.4**		**98.7**	**98.7**	**98.6**	86.9	86.5	75.9	73.8
**Lc 21.1**	**87.0**	**76.9**	**87.3**		**98.8**	**99.5**	87.0	86.5	76.3	73.3
Lactococcus lactis subsp. *lactis* ATCC 19435^{T}	**87.5**	**77.4**	**88.4**	**88.9**		**98.6**	87.0	86.6	76.0	73.3
Lactococcus lactis subsp. *lactis* biovar. *diacetylactis* GL2^{T}	**87.7**	**77.6**	**88.6**	**95.3**	**87.9**		86.5	86.5	75.8	73.0
Lactococcus cremoris NBRC 100676^{T}	32.7	32.5	32.5	32.8	32.7	31.6		98.0	76.0	72.7
Lactococcus cremoris subsp. *tructae* DSM 21502^{T}	32.0	31.6	31.8	31.8	31.7	31.4	83.6		75.9	72.7
Lactococcus allomyrinae 1JSPR-7^{T}	22.3	22.3	22.2	22.5	22.5	21.3	23.3	22.4		72.2
Lactococcus hircilactis DSM 28960^{T}	24.2	24.3	24.8	24.8	24.8	24.6	25.2	23.0	21.4	

**B** ANI / dDDH	**St 8.1**	**St 9.1**	**St 18.1**	**St 21.1**	*S. thermophilus* ATCC 19258^{T}	*S. vestibularis* NCTC 12167^{T}	*S. salivarius* NCTC 8618^{T}	*S. rubneri* DSM 26920^{T}	*S. infantarius* ATCC BAA-102^{T}
**St 8.1**		**99.4**	**99.9**	**99.4**	**98.8**	92.2	89.1	72.6	72.5
**St 9.1**	**94.5**		**99.4**	**99.9**	**98.8**	92.1	98.2	72.3	73.0
**St 18.1**	**99.8**	**94.6**		**99.4**	**98.7**	92.1	89.3	72.7	71.9
**St 21.1**	**94.8**	**99.7**	**94.6**		**98.6**	92.1	89.2	72.1	72.5
Streptococcus thermophilus ATCC 19258^{T}	**88.4**	**89.2**	**88.6**	**87.0**		92.2	89.4	72.7	71.1
Streptococcus vestibularis NCTC 12167^{T}	47.6	46.1	47.7	46.5	46.7		92.1	73.1	72.8
Streptococcus salivarius NCTC 8618^{T}	37.6	37.7	37.6	37.6	37.9	46.2		73.4	72.9
Streptococcus rubneri DSM 26920^{T}	26.2	26.3	26.2	26.3	26.5	29.3	27.4		71.1
Streptococcus infantarius ATCC BAA-102^{T}	25.5	25.0	25.6	25.0	26.6	25.8	25.7	21.7	

In bold, values with type strains above the species thresholds (95% for ANI and 70% for dDDH); underlined, the highest orthoANI and dDDH values.

Table S5. Presence of metabolic genes involved in growth in milk and the formation of flavour compounds (taste and aroma) in the genome of four GABA producer *Lactococcus lactis* strains.

Protein/description	Gene/operon	Function	Strain			
			LL5.5	LL14.4	LL19.3	LL21.1
**Proteases**						
ATP-dependent zinc metalloprotease	*ftsH*	Cleavage of transmembrane sequences, cell division	1	1	1	1
CAAX amino-terminal protease	-	-	-	1	1	-
Cell wall-bound caseinolytic proteinase	*prtP*	Digestion of milk caseins	1	1	1	-
Clp protease	*clpCEPX*	Turnover of cellular proteins	1	1	1	1
DegP/HtrA serine protease	*htrA*	Clearance of denatured or aggregated proteins from the inner membrane and periplasmic space	1	1	1	1
Late competence processing protease	*comC*	Processing of DNA during uptake	1	1	1	1
Lon-like protease with PDZ domain	-	Quality-control of proteins	1	1	1	1
RasP/YluC protease	-	-	1	1	1	1
Rhomboid serin protease	-	-	1	1	1	1
SOS-response repressor and protease	*lexA*	Autoproteolysis in SOS response	1	1	-	1
YmfH protease	*ymfH*		1	1	1	1
YrrO protease	*yrrO*	-	1	1	1	1
Zinc protease	-	-	1	1	1	1
**Peptidases**						
Aminopeptidase	*ypdF*	Hydrolyses Xaa-Pro bonds when Xaa is Ala, Asn or Met	1	1	1	1
Aminopeptidase C	*pepC*	General cysteine aminopeptidase	1	1	1	1
Dipeptidase	*pepQ*	Degradation of dipeptides	2	2	2	2
Glutamyl aminopeptidase	*pepA*	Aminopeptidase of Asp- and Glu- peptides	1	1	1	1
Lysyl aminopeptidase	*pepN*	General aminopeptidase	1	1	1	1
Methionine aminopeptidase	*pepM*	Release of Met from proteins and peptides	1	1	1	1
Multimodular transpeptidase-transglycosylase	-	Peptidoglycan biosynthesis, probably PBPs	3	3	3	3
Muramoyl tetrapeptide carboxypeptidase	-	-	1	1	1	1
Neutral endopeptidase	-	Endopeptidases	3	3	3	3
Oligoendopeptidase F	*pepF*	Hydrolyzes 7-17 amino acids long peptides	1	1	1	1
Peptidase E	*pepE*	Releases N-terminal Asp	-	1	-	-
Peptidase U32 family	-	-	1	1	1	1
Proline imidopeptidase	*pepD*	Cleaves dipeptides when Pro is at the carboxy-terminus	1			
Pyrrolidone-carboxylate peptidase	*pcp*	Removes the pyroglutamate from proteins and peptides	1	-	1	1
SprT-family zinc metallopeptidase	*sprT*	-	1	1	1	1
Tripeptide aminopeptidase	*pepT*	Amino degradation of tripeptides	1	1	1	1
Xaa-His dipeptidase	*pepV*	Hydrolysis of Xaa-His and general dipeptides	1	1	1	1
Xaa-Pro dipeptidyl peptidase	*pepX*	Release dipetides when Pro is at the second position	1	1	1	1
**Lactose, citrate, amino acid, and peptide transporters**						
Amino acid permease	*gabP*	Transport of amino acids	3	3	3	3
Arginine/ornithine antiporter	*arcD*	Transport arginine	2	1	2	3

Basic amino acid/glutamine ABC transporter	-	Transport of basic amino acids	2	2	2	2
Branched-chain amino acid transporter (Na-dependent)	*brnQ*	Transport of branched-chain amino acids	1	1	1	1
Cationic amino acid transporter	*yckB*	Transport of basic amino acids	1	1	1	1
Citrate transporter	*citQRP*	Citrate transporter	-	-	-	1
Cysteine ABC transporter	-	Transport of cysteine	2	2	2	2
Di-tripeptide/cation symporter DtpT	*dtpT*	Transport of di- and tripeptides	1	1	1	1
Glutamate/gamma-aminobutyrate antiporter	*gadC*	Transport glutamate	1	2	1	1
Glutamine ABC transporter	*glnHPQ*	Transport of basic amino acids	1	1	1	1
Lactose, galactose permease (GPH translocator)	*lacP*	Transport of lactose	1	-	1	1
Lactose-specific PTS system	*lacABC*	Transport and phosphorylation of lactose	1	1	1	1
Methionine ABC transporter	-	Transport of Met	1	1	1	1
Methionine/phosphonates ABC transporter	-	Transport of Met	1	1	1	1
Oligopeptide ABC transporter	*oppABCDF*	Transport of oligopeptides	1	1	1	1
**Aminotransferases and transaminases**						
Alanine transaminase	-	Arg synthesis, Ala, Asp, Glu metabolism	1	1	1	1
Aromatic amino acid aminotransferase gamma	*araT*	Transfer of α-amino groups between aromatic amino acids	1	1	1	1
Aspartate aminotransferase	-	Reversible transfer of an α-amino group between Asp and Glu	3	3	3	3
Branched-chain amino acid aminotransferase	*bcaT*	Cys, Val, Leu, Iso, Met, synthesis and metabolism, synthesis of secondary metabolites	1	1	1	1
Glutamine amidotransferase, class 1	*pfpI*	Removal of the ammonia group from glutamine	1	1	1	1
Glutamine-fructose-6-phosphate aminotransferase	*glmS*	Ala, Glu, metabolism, metabolic pathways	1	1	1	1
Histidinol-phosphate aminotransferase	-	Glu, His metabolic pathways	1	1	1	-
Multimodular transpeptidase-transglycosidase	-	-	3	3	3	3
N-acetyl-L,L-diaminopimelate aminotransferase	-	Ala, Asp, Glu, Gly, Ser, Thr metabolism, Lys degradation, pyruvate metabolism	1	1	1	1
N-acetylornithine aminotransferase	*rocD*	Arg biosynthesis, metabolic pathways, secondary metabolites	-	1	1	1
Phosphoserine aminotransferase	-	Gly, Ser, Thr, Cys, Met metabolism, secondary metabolites	1	1	1	1
Serine-pyruvate aminotransferase	-	Gly, Ser, Thr metabolism	1	1	1	1
**Dehydrogenases**						
2,3-butanediol dehydrogenase (R-S)	-	Biosynthesis of secondary metabolites	2	2	2	2
Acetaldehyde dehydrogenase	*adhE*	Fatty acids degradation, amino acids synthesis and degradation, synthesis of secondary metabolites	2	1	1	1
Alanine dehydrogenase	*ala-dh*	Ala, Asp, Glu metabolism, metabolic pathways	1	3	-	1
Alcohol dehydrogenase class III	*adh*	Glycolysis, fatty acid degradation, Tyr metabolism, pyruvate metabolism	1	1	1	1
Aspartate-semialdehyde dehydrogenase	-	Gly, Thr, Cys, Met metabolism, Lys synthesis	1	1	1	1
D-lactate dehydrogenase	*ldhD*	Synthesis of secondary metabolites	-	-	1	1
Homoserine dehydrogenase	-	Gly, Ser, Thr, Cys, Met metabolism, Lys synthesis, metabolic pathways	1	1	1	1
L-lactate dehydrogenase	*ldhL*	Glycolysis, Cys, Met metabolism, secondary metabolites	3	4	4	3
L-lactate dehydrogenase, Fe-S oxidoreductase	*ykgEFG*	Glycolysis, Cys, Met metabolism, secondary metabolites				1
**Lyases**						
Argininosuccinate lyase/synthase	-	Arg biosynthesis, Ala, Asp, Glu metabolism, Metabolic pathways, biosynthesis of secondary metabolites	1	1	1	1

Citrate lyase complex	*citCDEFG*	Formation of pyruvate from citrate	-	1	-	1
Cystathionine beta-lyase	*cglB*	Production of sulphur compounds	1	1	1	1
Cystathionine beta-synthase	*cbs*	Transulfuration from homocysteine to cystathionine	1	1	1	1
Cystathionine gamma-lyase	*cglA*	Gly, Ser, Met, Cys, Thr metabolism, metabolic pathways	1	1	1	1
Cystathionine gamma-synthase	*cgs*	Formation of cystathionine from cysteine	1	1	1	1
S-ribosylhomocysteine lyase	-	Cys, Met metabolism, metabolic pathways	1	1	1	1
**Esterases/lipases**						
Alpha/beta hydrolase (HAD family protein)	-	Lipid transport and metabolism	11	10	11	10
Carboxylesterase	*est*	Wide specificity acting on ester bonds	1	1	1	1
Esterase/lipase	-	Lipid transport and metabolism	1	1	1	1
Glycerophosphoryl diester phosphodiesterase	*glpQ*	Glycerophospholipid metabolism	2	2	2	2
Lipase/Acylhydrolase	-	-	2	2	2	2
Lysophospholipase/monoglyceride lipase	-	-	1	1	1	1
Phosphoesterase	-	Release of membrane-linked proteins with glycosylphosphatidylinositol anchors	2	2	1	2
Tributyrin esterase	-	Hydrolysis of C2-C16 fatty acids	1	1	1	1

Highlighted in yellow are differences in gene content between *L. lactis* strains and in green species-specific genes as compared to the *Streptococcus thermophilus* strains in Table S2.

Table S6. Presence of metabolic genes involved in the growth in milk and formation of flavour compounds (taste and aroma) in the genome of four GABA producer *Streptococcus thermophilus* strains.

Protein/description	Gene/operon	Function	Strain			
			St 8.1	St 9.1	St18.1	St 21.1
**Proteases**						
ATP-dependent zinc metalloprotease	*ftsH*	Cleavage of transmembrane sequences, cell division	1	1	1	1
CAAX amino-terminal protease	-	-	1	-	1	-
CAAX membrane-bound protease	-	-	1	1	1	1
Cell wall-bound caseinolytic proteinase	*prtS*	Digestion of milk caseins	1	-	1	-
Clp protease	*clpCEPX*	Turnover of cellular proteins	1	1	1	1
DegP/HtrA serine protease	*htrA*	Clearance of denatured or aggregated proteins from the inner-membrane and periplasmic space	1	1	1	1
Late competence processing protease	*comC*	Processing of DNA during uptake	1	1	1	1
Lon-like protease with PDZ domain	-	Quality-control of proteins	1	1	1	1
RasP/YluC protease	-	-	1	1	1	1
YmfH protease	*ymfH*		-	1	-	1
YrrO protease	*yrrO*	-	1	1	1	1
Zinc protease	-	-	1	1	1	1
**Peptidases**						
Aminopeptidase	*ypdF*	Hydrolyses Xaa-Pro bonds when Xaa is Ala, Asn or Met	1	1	1	1
Aminopeptidase C	*pepC*	General cysteine aminopeptidase	1	1	1	1
Aminopeptidase S	*pepS*	Cleaves of preference Leu, Val, Phe, and Tyr	1	1	1	1
Dipeptidase	*pepQ*	Degradation of dipeptides	-	1	-	1
Glutamyl aminopeptidase	*pepA*	Aminopeptidase of Asp- and Glu- peptides	1	1	1	1
Lysyl aminopeptidase	*pepN*	General aminopeptidase	1	1	1	1
Methionine aminopeptidase	*pepM*	Release of Met from proteins and peptides	1	1	1	1
Neutral endopeptidase O	*pepO*	Endopeptidase	1	1	1	1
Oligoendopeptidase F	*pepF*	Hydrolyzes 7-17 amino acids long peptides	1	1	1	1
Peptidase U32 family	-	-	1	1	1	1
Proline imidopeptidase	*pepD*	Cleaves dipeptides when Pro is at the carboxy-terminus	1	1	1	1
Tripeptide aminopeptidase	*pepT*	Amino degradation of tripeptides	1	1	1	1
Xaa-His dipeptidase	*pepV*	Hydrolysis of Xaa-His and general dipeptides	1	1	1	1
Xaa-Pro dipeptidyl peptidase	*pepX*	Release dipetides when Pro is at the second position	1	1	1	1
**Lactose, amino acid, and peptide transporters**						
Amino acid permease	*gabP*	Transport of amino acids	1	1	1	1
Amino acid permease	*ydaO*	Transport of amino acids	1	1	1	1
Basic amino acid/glutamine ABC transporter	-	Transport of basic amino acids	1	1	1	1
Branched-chain amino acid transporter	*livFGHJM*	Transport of Leu, Iso, Val	1			
Branched-chain amino acid transporter	*azlC*	-	1			
Branched-chain amino acid transporter (Na-dependent)	*brnQ*	Transport of branched chain amino acids	1	1	1	1

Cationic amino acid transporter	*yckB*	Transport of basic amino acids	1	1	1	1
Cysteine ABC transporter	-	Transport of cysteine	1	2	2	1
Di-tripeptide/cation symporter DtpT	*dtpT*	Transport of di- and tripeptides	1	1	1	1
Glutamate/gamma-aminobutyrate antiporter	*gadC*	Transport glutamate	1	1	1	1
Glutamine ABC transporter	*glnHPQ*	Transport of basic amino acids	1	1	1	1
Lactose, galactose permease (GPH translocator)	*lacP*	Transport of lactose	1	1	1	1
Methionine ABC transporter	-	Transport of Met	1	1	1	1
Oligopeptide ABC transporter	*oppABCDF*	Transport of oligopeptides	1	1	1	1
Serine/threonine Na+ symporter	*sstT*	Transport of Ser, Thr	1	1	1	1
Sodium/glycine symporter GlyP	*glyP*	Transport of Gly	1	1	1	1
**Aminotransferases and transaminases**						
Alanine transaminase	-	Arg synthesis, Ala, Asp, Glu metabolism	1	1	1	1
Aromatic amino acid aminotransferase gamma	*araT*	Transfer of α-amino groups between aromatic amino acids	1	1	1	1
Aspartate aminotransferase	*aspAT*	Reversible transfer of a α-amino group between Asp and Glu	2	2	2	2
Branched-chain amino acid aminotransferase	*bcaT*	Cys, Val, Leu, Iso, Met, synthesis and metabolism, synthesis of secondary metabolites	1	1	1	1
Glutamine amidotransferase, class 1	*pfpI*	Removal of the ammonia group from glutamine	1	2	1	2
Glutamine-dependent 2-keto-4-methylthiobutyrate transaminase	-	Cys, Met metabolism, metabolic pathways	1	2	1	2
Glutamine-fructose-6-phosphate aminotransferase	*glmS*	Ala, Glu, metabolism, metabolic pathways	1	1	1	1
Histidinol-phosphate aminotransferase	-	Glu, His metabolic pathways	1	1	1	1
Multimodular transpeptidase-transglycosidase	-	-	2	2	2	2
N-acetyl-L,L-diaminopimelate aminotransferase	-	Ala, Asp, Glu, Gly, Ser, Thr metabolism, Lys degradation, pyruvate metabolism	1	1	1	1
N-acetylornithine aminotransferase	*rocD*	Arg biosynthesis, metabolic pathways, secondary metabolites	1	1	1	1
Phosphoserine aminotransferase	-	Gly, Ser, Thr, Cys, Met metabolism, secondary metabolites	1	1	1	1
**Dehydrogenases**						
2,3-butanediol dehydrogenase (R-S)	-	Biosynthesis of secondary metabolites	1	1	1	1
Alanine dehydrogenase	*ala-dh*	Ala, Asp, Glu metabolism, metabolic pathways	1	1	1	1
Aspartate-semialdehyde dehydrogenase	-	Gly, Thr, Cys, Met metabolism, Lys synthesis	1	1	1	1
Glutamate dehydrogenase (NADP-specific)	*gdhA*	Arg synthesis, Ala, Asp, metabolism, metabolic pathways	1	1	1	1
Homoserine dehydrogenase	-	Gly, Ser, Thr, Cys, Met metabolism, Lys synthesis, metabolic pathways	1	1	1	1
L-lactate dehydrogenase	*ldhL*	Glycolysis, Cys, Met metabolism, secondary metabolites	2	2	2	2
**Lyases**						
Argininosuccinate lyase/synthase	-	Arg biosynthesis, Ala, Asp, Glu metabolism, Metabolic pathways, biosynthesis of secondary metabolites	1	1	1	1
Cystathionine beta-lyase	*cglB*	Production of sulphur compounds	1	1	1	1
Cystathionine beta-synthase	*cbs*	Transulfuration from homocysteine to cystathionine				
Cystathionine gamma-lyase	*cglA*	Gly, Ser, Met, Cys, Thr metabolism, metabolic pathways	1	1	1	1
Cystathionine gamma-synthase	*cgs*	Formation of cystathionine from cysteine	1	1	1	1
D-histidine ammonia-lyase	-	Gly, Ser, Thr metabolism, D-Amino acid metabolism, metabolic pathways	1	1	1	1

S-ribosylhomocysteine lyase	*luxS*	Cys, Met metabolism, metabolic pathways	1	1	1	1
**Esterases/lipases**						
Alpha/beta hydrolase (HAD family protein)	-	Lipid transport and metabolism	7	9	10	8
Esterase/lipase	-	Lipid transport and metabolism	1	1	1	1
Phosphoesterase	-	Release of membrane-linked proteins with glycosylphosphatidylinositol anchors	1	1	1	1
Thiosterase	-	Fatty acid metabolism	3	3	3	3
Tributyrin esterase	-	Hydrolysis of C2-C16 fatty acids	1	1	1	1

Highlighted in yellow are differences in gene content between *S. thermophilus* strains and in green species-specific genes as compared to the *Lactococcus lactis* strains in Table S1.

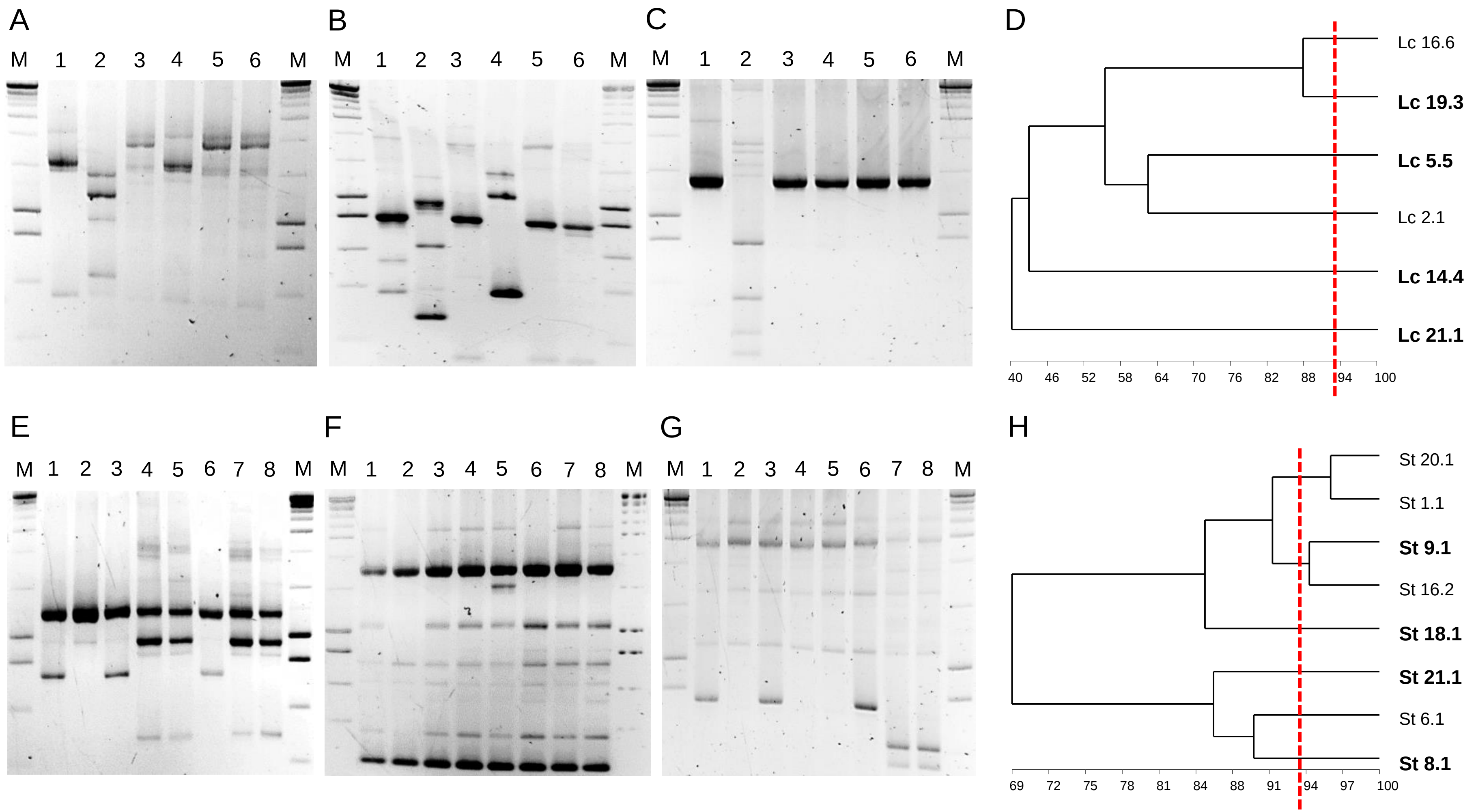

Figure S1. RAPD and rep-PCR typing profiles of *Lactococcus lactis* (A, B, C) and *Streptococcus thermophilus* (E, F,G) GABA-producing strains obtained with primers M13 (A, E), BoxA2R (B, F), and OPA18 (C, G), and dendrogram of similarity (D, H) of the combined typing profiles expressed by the Jaccard similarity coefficient (in %). Clustering was performed by the unweighted pair group method using arithmetic averages (UPGMA). The red line marks the repeatability of the typing technique (93%). In bold, strains selected for further phenotypic testing and genome sequencing.

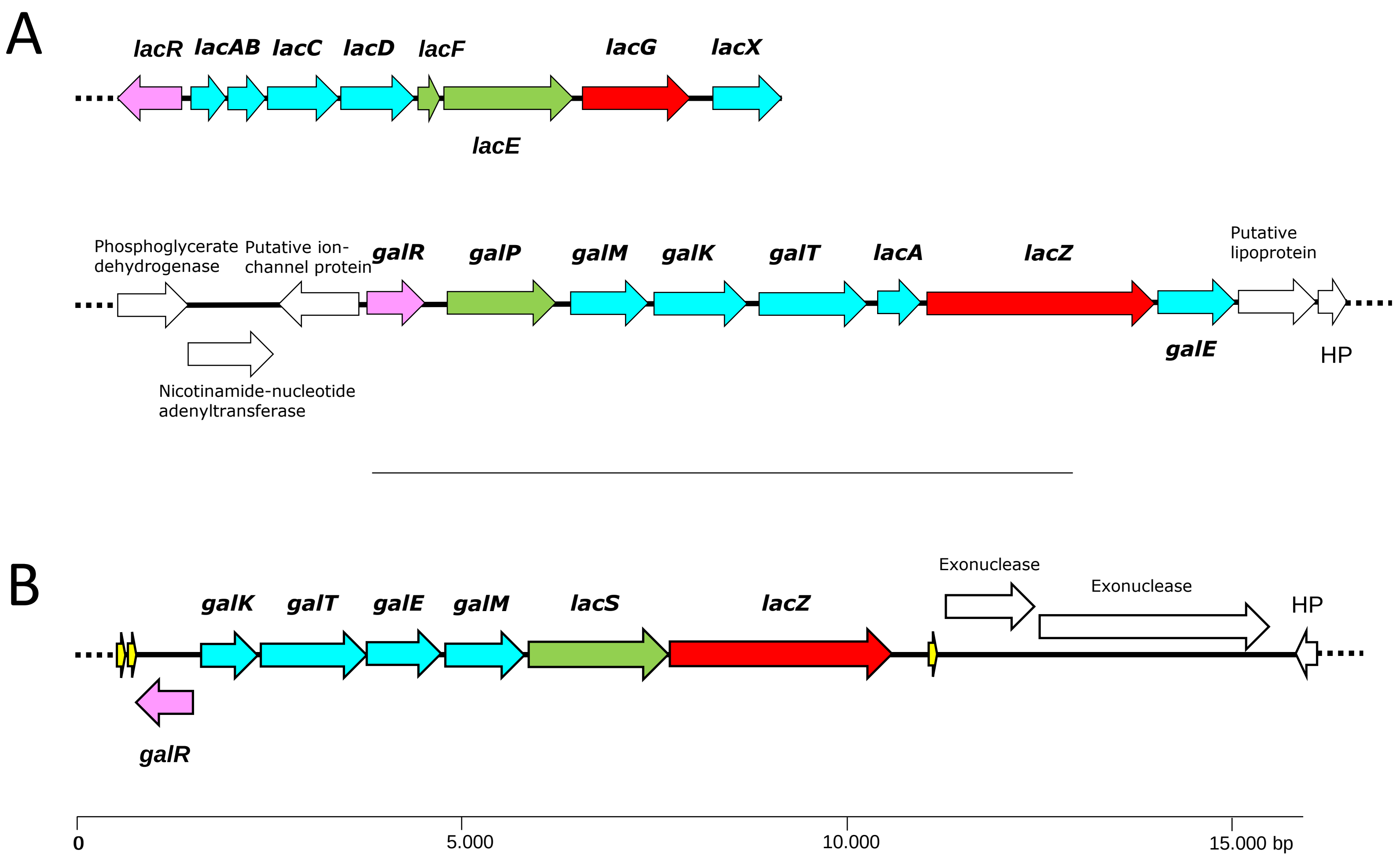

Figure S2. Genetic organization of the lactose/galactose operons found in the genome of *Lactococcus lactis* (A) and *Streptococcus thermophilus* (B) strains. Colour code: in red, genes coding for β-galactosidase (*lacZ*) or phospho-β-galactosidase (*lacG*); in green, genes involved in transport (*lacFE*, PEP-PTS components; *galP*, galactose permease; *lacS*, lactose/galactose permease); in purple, genes coding for regulatory proteins (*lacR*, *galR*); in pale blue, other genes of sugar metabolism (*lacAB*, galactose isomerase; *lacC*, tagatose-6-phosphate kinase; *lacD*, tagatose-1,6-phosphate aldolase; *galE*, UDP-glucose-4-epimerase; *galK*, galactokinase; *galM*, aldose-1-epimerase; *galT*, Galactose-1-phosphate uridyltransferease; *lacA*, galactoside acetyltransferase); in yellow, partial genes coding for transposases; in white, genes encoding proteins of other systems or ORFs encoding hypothetical proteins. Dotted lines indicate that the contig extends beyond the depicted area.

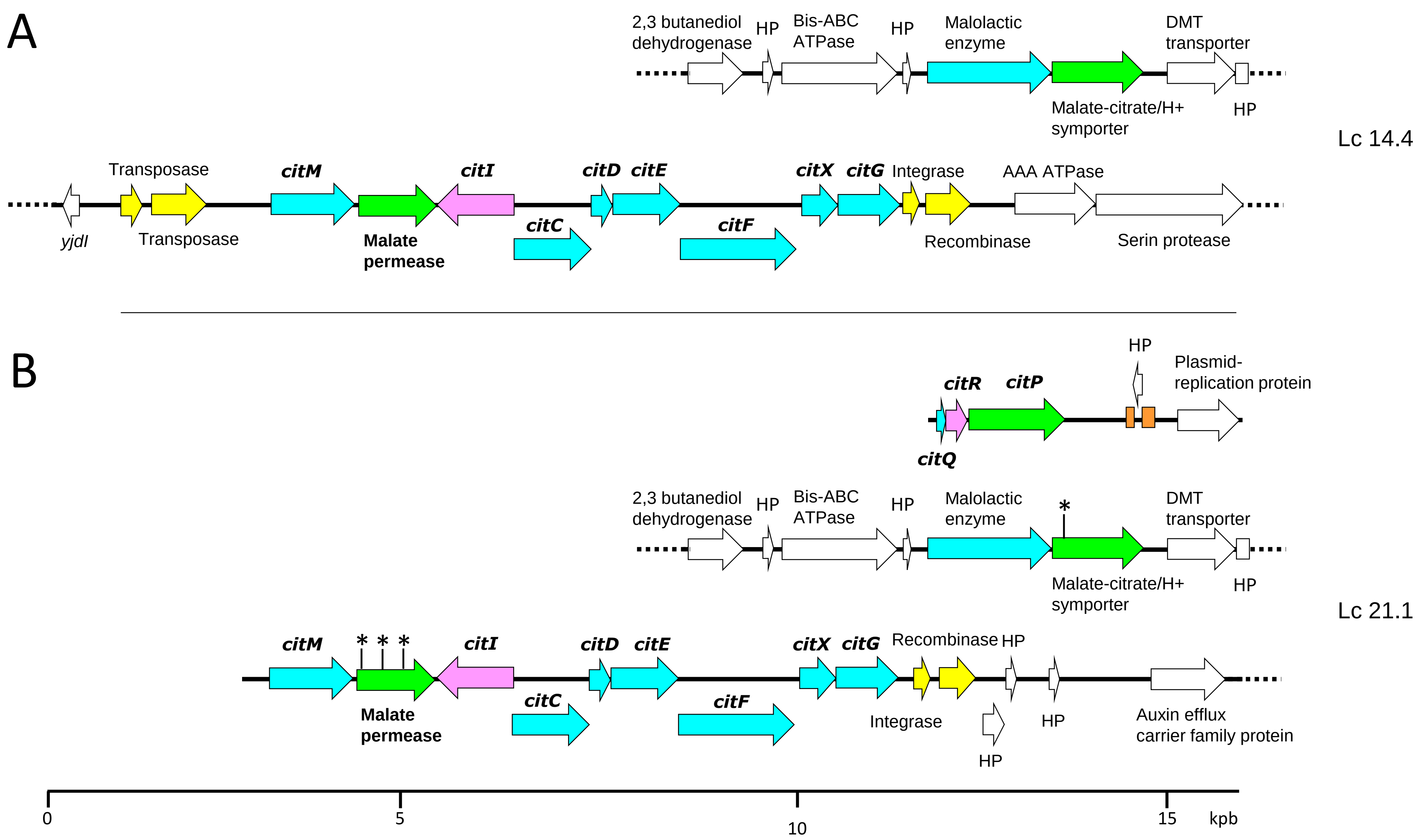

Figure S3. Genetic organization of citrate gene clusters found in the genome of *L. lactis* Lc 14.4 (A) and Lc 21.1 (B). Colour code: in pale blue, genes of the citrate lyase operon (*citM-citCDEFXG*) and malolactic enzyme (*mleA*); in green, genes involved in transport, including the citrate permease, *citP,* and two malate-citrate/H+ symporters; in purple, genes encoding regulatory proteins; in yellow, genes coding for mobilization proteins; in white, other genes or genes encoding hypothetical proteins. Boxes indicate DNA repeats (brown) or N-terminal partial genes (white). Asterisks mark mutations disrupting the ORFs. Broken lines indicate that the contigs extend beyond these positions.

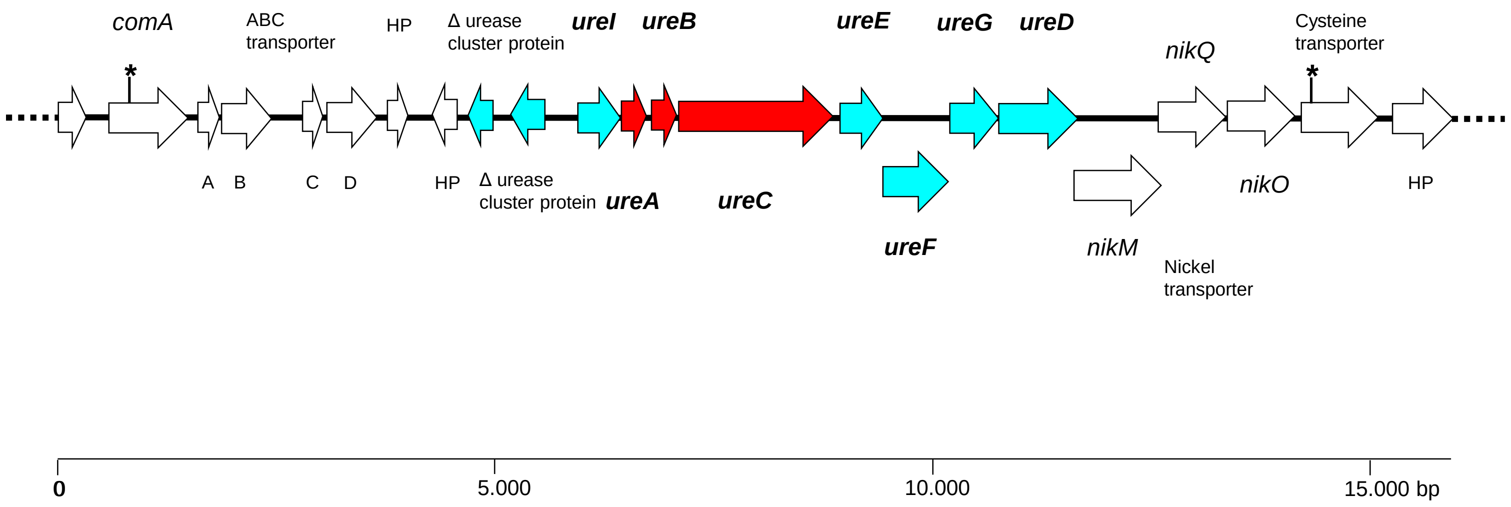

Figure S4. Genetic organization of the urease operon in the genome of *Streptococcus thermophilus* strains. Color code: in red, urease catalytic genes (*ureA, ureB,* and *ureC*); in pale blue genes, accessory genes; in white, genes encoding hypothetical proteins or proteins from other systems. Dotted lines indicate that the contig extends beyond the depicted area. Asterisks mark mutations disrupting the ORFs.
